# Performance evaluation of an AI-based preoperative planning software application for automatic selection of pedicle screws based on computed tomography images

**DOI:** 10.3389/fsurg.2023.1247527

**Published:** 2023-09-11

**Authors:** Shanhang Jia, Yuanzhi Weng, Kai Wang, Huan Qi, Yuhua Yang, Chi Ma, Weijia William Lu, Hao Wu

**Affiliations:** ^1^ Department of Neurosurgery, Xuanwu Hospital, Capital Medical University, Beijing, China; ^2^ Spine Center, China International Neuroscience Institute (CHINA-INI), Beijing, China; ^3^ Department of Orthopaedics and Traumatology, Li Ka Shing Faculty of Medicine, The University of Hong Kong, Hong Kong, Hong Kong SAR, China; ^4^ Department of Orthopaedics and Traumatology, The University of Hong Kong-Shenzhen Hospital, Shenzhen, China; ^5^ Department of Pharmaceutical Materials Science and Translational Medicine, Shenzhen Institute of Advanced Technology, Chinese Academy of Sciences (CAS), Shenzhen, China

**Keywords:** pedicle screw, AI, surgical planning, insertion accuracy, computed tomography

## Abstract

**Introduction:**

Recent neurosurgical applications based on artificial intelligence (AI) have demonstrated its potential in surgical planning and anatomical measurement. We aimed to evaluate the performance of an AI planning software application on screw length/diameter selection and insertion accuracy in comparison with freehand surgery.

**Methods:**

A total of 45 patients with 208 pedicle screw placements on thoracolumbar segments were included in this analysis. The novel AI planning software was developed based on a deep learning model. AI-based pedicle screw placements were selected on the basis of preoperative computed tomography (CT) data, and freehand surgery screw placements were observed based on postoperative CT data. The performance of AI pedicle screw placements was evaluated on the components of screw length, diameter, and Gertzbein grade in comparison with the results achieved by freehand surgery.

**Results:**

Among 208 pedicle screw placements, the average screw length/diameters selected by the AI model and used in freehand surgery were 48.65 ± 5.99 mm/7.39 ± 0.42 mm and 44.78 ± 2.99 mm/6.1 ± 0.27 mm, respectively. Among AI screw placements, 85.1% were classified as Gertzbein Grade A (no cortical pedicle breach); among free-hand surgery placements, 64.9% were classified as Gertzbein Grade A.

**Conclusion:**

The novel AI planning software application could provide an accessible and safe pedicle screw placement strategy in comparison with traditional freehand pedicle screw placement strategies. The choices of pedicle screw dimensional parameters made by the model, including length and diameter, may provide potential inspiration for real clinical discretion.

## Introduction

Strategies for pedicle screw placement mainly focus on two components: screw dimensions and screw trajectory. For screw trajectory, there are many guidelines, including empirical guidance and radiological instruction. Specific considerations include entry point, parallelization between the screw and superior endplate, mediolateral inclination, and suitable insertion depth (1–3). Regarding insertion depth, the popular AO reference indicates that the inclination angle should be based on vertebral rotation, which may help to avoid penetration of the vertebral anterior cortex (3).

Regarding screw dimensions, published papers and reviews have mainly focused on the anatomical dimension of the pedicle; suggestions are variable, depending on specific surgical considerations (4). For thoracic pedicle screw insertion, the ideal screw diameter is approximately 80% of the pedicle diameter (5). Takeshita et al. indicate that the screw diameter should be less than 125% of the pedicle width for scoliotic patients (6). A cortical safety margin of 0.5 mm has been reported in previous research on thoracic spinal screw insertion (7, 8). Regarding lumbar spinal pedicle insertion, an investigation with over 400 patients showed that there is a significant difference between different races in terms of lumbar pedicle width, which is a necessary consideration in further screw insertion surgeries (9). A paper published in 2019 indicates that screw length should be less than 75%–85% of the vertebral body up to the specific segments (10). However, the available guidance on pedicle/screw diameter ratio, cortical margin, and vertebral body length remains complicated and impractical for preoperative planning. Doctors still need to measure the pedicle diameter before they determine the screw dimensions. In relation to the other component, screw trajectory, it is difficult to incorporate trajectory considerations into the above-described process. In addition, the time and labor costs may become burdens of its application, which may result in a preference for purely empirical determination of screw size.

Recent neurosurgical applications based on artificial intelligence (AI) have demonstrated their potential in surgical planning and anatomical measurement. Siemionow et al. trained an automatic pedicle screw placement model based on vertebral anatomical features using a neural network (11). Vijayan et al. established a pedicle screw planning model based on reference trajectories labeled by a spinal surgeon and by anatomical measurement (12). One automatic AI-based screw trajectory planning algorithm, reported previously by us, was found to be capable of generating the optimal pedicle screw path, with higher bone mineral density (BMD) and pullout force (POF) than the AO reference path (13). To the authors’ knowledge, there no study has been conducted to compare AI and freehand surgery in terms of screw length and diameter selection, with insertion accuracy considerations.

In this investigation, we present and evaluate the performance of an AI planning software application in screw length/diameter selection and insertion accuracy in comparison with freehand surgery. Our research hypothesis was that the preoperative AI-based surgical planning software could provide better screw dimensions (with longer lengths and wider diameters being selected), with greater insertion accuracy than that achieved in freehand surgery.

## Methods

### Patient demographics

Patients who underwent freehand thoracolumbar internal fixation surgeries were included in this investigation. All enrolled patients had lumbar degenerative diseases or thoracic kyphosis. All surgeries were completed by the same surgical team between June and December 2022 in the neurosurgery department at Xuanwu Hospital. The exclusion criteria were as follows: (1) patients with bone tumor, ankylosing spondylitis, diffuse idiopathic skeletal hyperostosis, rheumatoid arthritis, tuberculosis, or secondary osteoporosis; (2) patients undergoing reoperation after initial internal fixation due to postoperative complications; and (3) patients with severe scoliosis before surgery (Cobb angle >45°).

### Computed tomography scanning

Preoperative computed tomography (CT) images of each patient were generally obtained during the 2 days before surgery, while postoperative CT images were normally collected within 3 days after surgery. All CT images were generated in Digital Imaging and Communications in Medicine (DICOM) format using Revolution CT from GE MEDICAL SYSTEMS. The slice thickness of the CT images was 0.625 mm.

### Screw selection by the deep learning model and by surgeons

A novel deep learning model, based on a self-developed 3D-Unet algorithm, was utilized for preoperative pedicle screw planning. The primary version of this model has been published in a previous paper, which mainly focused on comparing the pullout force of the AO insertion method and that of the AI model. In the present study, the aforementioned self-developed screw planning AI model (13) was incorporated into a complete AI surgical planning software application, and its functionality was also improved. Preoperative CT images of all patients in DICOM format were imported into the AI surgical planning software application (Surgiplan AI) for generation of an AI-based internal fixation plan. After loading of the CT images, segments needing pedicle screw insertion were labeled for AI-based confirmation of the operation. The entry point of screws was limited to a circular region where the lateral border of the superior articular facet and middle transverse process intersected. Subsequently, the AI model calculated the default screw dimensions and trajectories and placed the screw within the vertebral pedicle. It must be noted that the longest possible screw (not exceeding the length between the entry point and vertebral front end) and the thickest possible (not exceeding 90% of the pedicle width) for the given pedicle were selected in AI-based planning, as screw length and diameter have a positive correlation with the pullout force of the screw (14). Additionally, a safety interval (≥1 mm) was retained in the inferior/medial pedicle walls. Finally, the pedicle screw was required to never violate the superior/inferior endplates.

**Figure 1:**
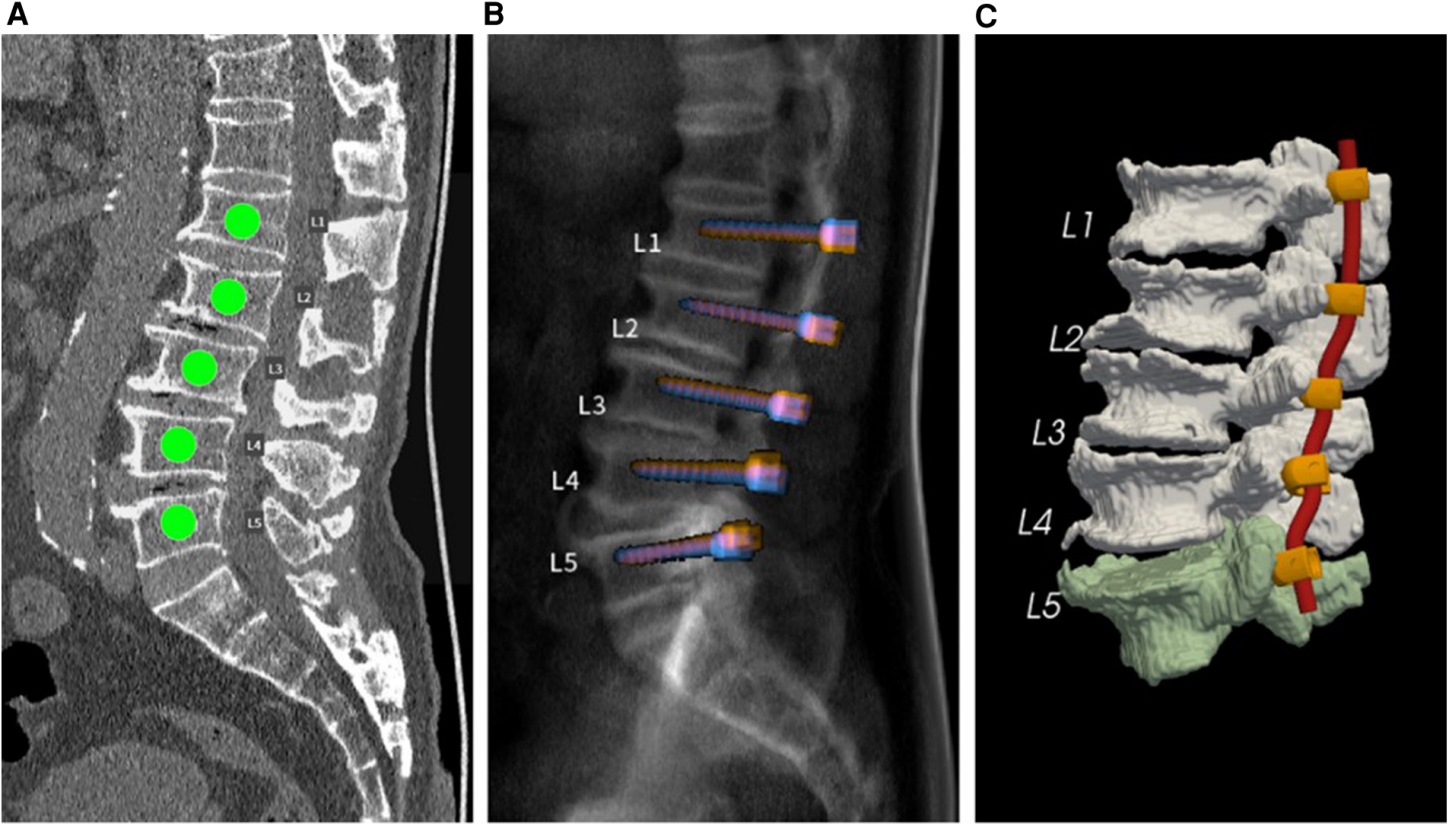
(**A**) Spinal segments requiring screw insertion. (**B**) Simulated sagittal X-ray view of default pedicle screw planning. (**C**) Three-dimensional view of the vertebral segmentation model with planned screw-rod systems.

Regarding screw trajectories, the default screw trajectories were those with the highest BMD and POF. After the default planning calculation was performed, the outcome was displayed in an interactive user interface for further analysis, such as evaluation of insertion accuracy. Figure 1 shows several interfaces in the AI preoperative planning software application, including labeling, X-ray view, and three-dimensional view.

The screw dimensions used in real freehand surgeries were determined by the surgeons based on experience. Surgeons selected the optimal screw based on simple observation and measurements provided on preoperative CT images; the reference guidelines were the AO spine surgery reference.

### Insertion accuracy evaluation

**Figure 2:**
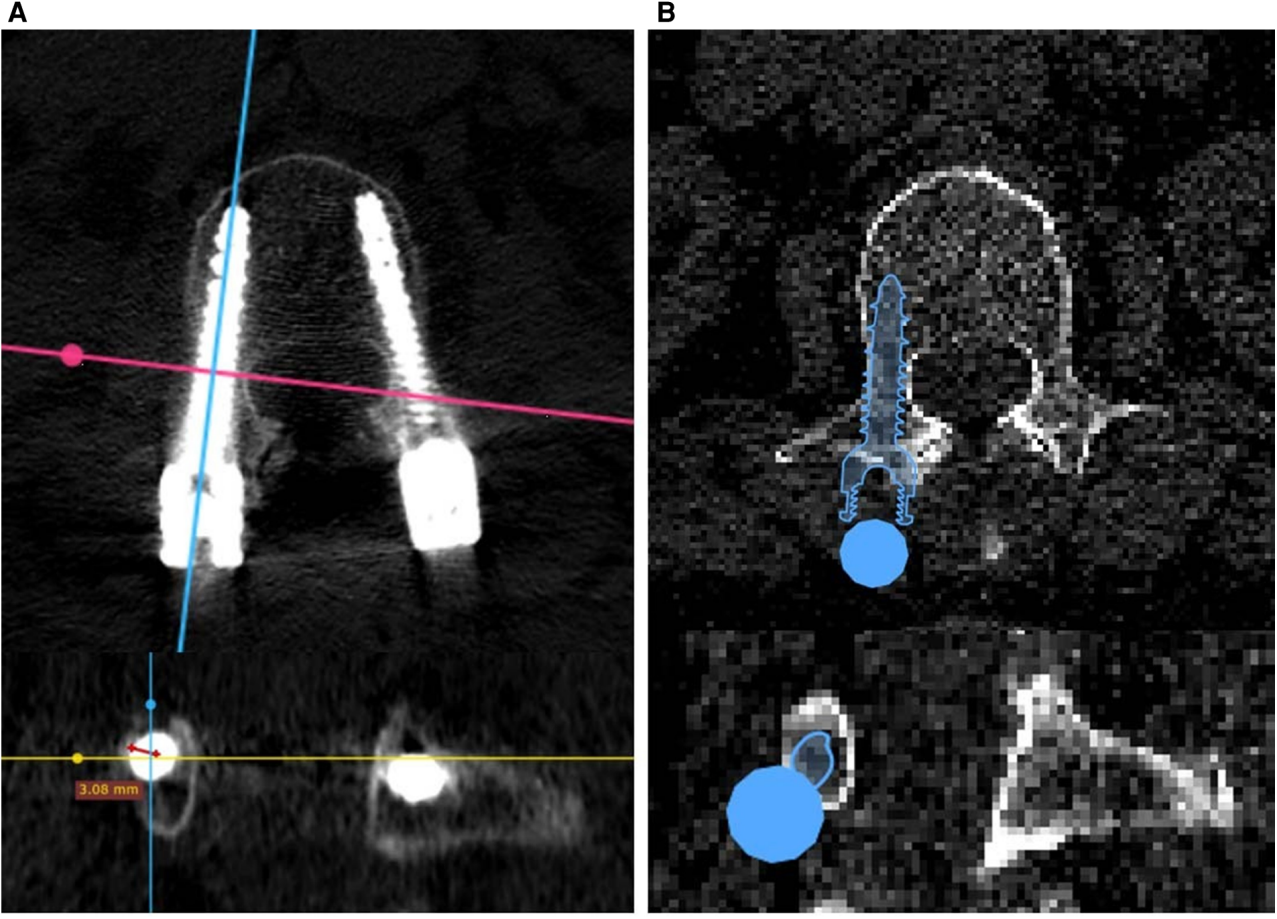
Gertzbein–Robbins evaluation of (**A**) freehand surgeries based on postoperative CT images and (**B**) AI planning results based on preoperative CT images.

The insertion accuracy of the results of the AI-based planning and freehand surgeries was evaluated using the Gertzbein–Robbins (GR) classification, consisting of grades A–E: grade A means there is no pedicle breach; grade B means the pedicle breach depth is <2 mm; and grades C, D, and E mean the pedicle breach depth is <4, <6 mm, and >6 mm, respectively. The insertion accuracy of freehand surgical results was evaluated using the Radiant DICOM viewer software package, specifically its three-dimensional multiplanar reconstruction mode. By adjusting the viewing direction of CT planes, screw-axis cross-sectional planes could be displayed for GR classification. The insertion accuracy of the AI-based planning results was directly evaluated using the Surgiplan AI software application, as the screw-axis cross-sectional mode could easily be switched on or off. Figure 2 shows examples of GR evaluation for freehand surgeries and AI-based planning.

### Statistics

Statistical analysis was carried out using the GraphPad PRISM software package. Paired *t*-tests were conducted to compare screw length and diameter between the AI model group and the freehand group for both the left and right sides, with *p* < 0.05 taken to represent a statistically significant difference. Fisher's exact test was conducted to compare GR grades between the AI model group and the freehand group, with *p* < 0.0001 was taken to represent a significant difference.

## Results

A total of 208 screw placements across 45 patients (15 men, 30 women) were examined in this study. Most segments for internal fixation were L4 and L5, covering a total of 79 of the 104 segments (Table 1). On both the left and right sides, the AI model was found to generate relatively longer (left: 49.28 ± 5.9 mm, right: 48.03 ± 6.02 mm) and thicker (left: 7.38 ± 0.41 mm, right: 7.39 ± 0.43) screw placements than those used in freehand surgeries (Table 2). The screw lengths and diameters in the freehand surgeries were 44.81 ± 3.17/6.1 ± 0.27 mm for the left side and 44.76 ± 2.81/6.1 ± 0.27 mm for the right side. Paired *t*-tests showed that there was a significant difference between the freehand group and the AI group on both length and diameter (all corresponding *p* values < 0.05).

**Table 1.** Patient information.

Number of patients	45
Age (years)	67.58 ± 8.27
Male	15
Female	30
BMI (kg/m^2^)	26.09 ± 3.51
Screw insertion segments	
T10	2
T11	2
T12	3
L1	2
L2	6
L3	10
L4	40
L5	39
Total segments	104
Screw placements	208

BMI, body mass index.

Values are given as *n* or mean ± SD.

**Table 2.** Screw length and diameter information.

Screw insertion	Screw length (mm)	Screw length range (mm)	Screw diameter (mm)	Screw diameter range (mm)
Freehand left	44.81 ± 3.17	40–60	6.1 ± 0.27	5–6.5
Freehand right	44.76 ± 2.81	40–50	6.1 ± 0.27	5–6.5
Freehand total	44.78 ± 2.99	40–60	6.1 ± 0.27	5–6.5
AI left	49.28 ± 5.9	40–65	7.38 ± 0.41	5–7.5
AI right	48.03 ± 6.02	30–60	7.39 ± 0.43	5–7.5
AI total	48.65 ± 5.99	30–65	7.39 ± 0.42	5–7.5

Values are given as mean ± SD unless otherwise indicated.

Regarding insertion accuracy, among the 208 screw insertions proposed by the AI model, 177 screw placements (85.10%) were classified as GR grade A (no cortical pedicle breach); and of all placements made in freehand surgeries, 135 (64.9%) were classified as GR grade A. Only four placements made by the AI model were classified as GR grade C, and the corresponding number for the freehand surgeries was 15. There were no screw placements with GR grades D or E among the AI model placements, but two screw placements with GR grade D were identified in the freehand surgery placements (Table 3). Table 4 compares the number of cases with GR grade A between the two groups. Fisher's exact test showed that the AI group had more cases achieving GR grade A (*p* < 0.0001).

**Table 3.** Summary of Gertzbein grading of freehand and AI-based screw placements.

Gertzbein grade	Freehand left	Freehand right	AI left	AI right
A	73	62	94	83
B	27	29	9	18
C	4	11	1	3
D	0	2	0	0
E	0	0	0	0
Total A-grade proportion by side	70.19%	59.62%	90.38%	79.81%
Total A-grade proportion by screw	64.90%		85.10%	

**Table 4.** Results of Fisher's exact test comparing AI and freehand results on the proportion of A grades achieved.

Gertzbein grade	A	Other
AI	177	31
Freehand	135	73
*p*-value (Fisher's exact test)	<0.0001 (****)	

**** means significant difference.

## Discussion

Longer and thicker screws have been proven to strengthen the pullout force (14); therefore, selection of an appropriate screw length and diameter affects the prognosis of an internal fixation. In existing reports on clinical operations and academic research, screw selection based on experience or simple measurements is expensive in terms of time and labor, but the final choice may not be the optimal option, as this method of screw selection is still manual and approximate.

In the present study, the AI model demonstrated better performance on screw length and diameter selection in comparison with selection based on freehand experience. The average planning time requirement for a single CT DICOM image was just 1–3 min; considering a surgeon's daily workload, this time cost was regarded as acceptable. The benefit becomes more meaningful in consideration of the fact that preoperative screw selection time is approximately 5–10 min when selection is performed manually. Additionally, the AI-based screw placements were validated as having better insertion accuracy was better than that achieved in freehand postoperative results, which means that the screw dimensional parameters determined using the AI model have a guarantee of safety. Furthermore, real-time monitoring of screw insertion accuracy during preoperative planning played a significant role, as doctors could confirm whether the screw selected by the AI model caused a vertebral breach or not, especially using the screw-axis reconstruction plane and three-dimensional view. The AI results should be viewed as a providing a rapid reference for determination of screw length and diameter determination. The model is not perfect; in our investigation, approximately 15% of the results still involved pedicle breach, and surgeons would still need to further confirm the validity of the determination and perform manual modifications where necessary.

The benefit of AI preoperative planning of screw length and diameter was that it supplied doctors with an upper limit for anti-loosening performance with regard to screw dimensional strategy. The AI plan may not be the same as what is finally implanted during the internal fixation, but it provides reasonable support for the selection of longer or thicker screws in comparison with those selected based on experience or using simple measurements. The insertion accuracy achieved by the AI model, on the other hand, solidified its validity, as pedicle breach is a common and conventionally used clinical standard. The AI software application was found to represent a practical and transferable advancement, as it improved the prognosis of internal fixation through a highly efficient and user-friendly method. With regard to intraoperative implementation, several possible methodologies, including surgical guides and surgical robots, have been published. For surgical guides, one previous study has proved that the average horizontal deviation between the preoperative plan and the postoperative result could be smaller than 1 mm (15). For surgical robots, existing papers have demonstrated that these can achieve a pedicle screw placement accuracy of approximately 95% (16–18). Therefore, implementation of the AI preoperative plan, including dimensions and trajectories, will not be a barrier to its clinical application.

In comparison with similar AI models presented in existing papers, the results of our model, in terms of the selection of longer and thicker screws, were unique and represent a significant advancement. Most existing models have only focused on automatic screw placement based on a doctor's experience with screw trajectory and vertebral anatomy (12, 19). The results of these models do not represent the upper limit of an accurate anti-loosening solution. Furthermore, the three-dimensional view and screw-axis plane shown in our application could enable more effective confirmation of planning validity. The incorporation of bone mineral density and estimated pullout force are other advantages of our model; screw length and diameter are constrained by the bone mineral density associated with the screw trajectory (13), a factor that has been neglected by most existing models. The validity and reliability of this AI model were demonstrated by comparing its selection results and insertion accuracy with real clinical postoperative results.

There were still some limitations to this investigation. First, most of the patients included were female (*n* = 30/45); some previous papers have indicated that there are differences between male and female spinal pedicle widths and other spinal topological features (20). In addition, the sample was still relatively small. Future studies may need to validate the screw selection performance in a larger number of patients of different genders, which may provide a better evaluation of this AI model. Second, patients with scoliosis were not included in this study, as only CT scans of normally aligned patients were used during the AI model training process. However, further research examining screw dimension and path generation for scoliotic patients may be an interesting direction for the study of this AI model.

## Conclusion

The novel AI planning software described here could provide an accessible and safe pedicle screw placement strategy in comparison with traditional freehand pedicle screw placement strategies. The choices of pedicle screw dimensional parameters made by the model, including length and diameter, may provide potential inspiration for real clinical discretion.

## Data Availability

The raw data supporting the conclusions of this article will be made available by the authors, without undue reservation.
